# Comparison of Polysomnography, Single-Channel Electroencephalogram, Fitbit, and Sleep Logs in Patients With Psychiatric Disorders: Cross-Sectional Study

**DOI:** 10.2196/51336

**Published:** 2023-12-13

**Authors:** Keita Kawai, Kunihiro Iwamoto, Seiko Miyata, Ippei Okada, Hiroshige Fujishiro, Akiko Noda, Kazuyuki Nakagome, Norio Ozaki, Masashi Ikeda

**Affiliations:** 1 Department of Psychiatry Nagoya University Graduate School of Medicine Nagoya Japan; 2 Department of Biomedical Sciences Chubu University Graduate School of Life and Health Sciences Kasugai Japan; 3 Department of Psychiatry National Center of Neurology and Psychiatry Kodaira Japan; 4 Pathophysiology of Mental Disorders Nagoya University Graduate School of Medicine Nagoya Japan

**Keywords:** consumer sleep-tracking device, polysomnography, portable sleep EEG monitor, electroencephalography, EEG, psychiatric disorders, sleep logs, sleep state misperception, polysomnography, sleep study, wearable, psychiatric disorder, sleep, disturbances, quality of sleep, Fitbit, mHealth, wearables, psychiatry, electroencephalogram

## Abstract

**Background:**

Sleep disturbances are core symptoms of psychiatric disorders. Although various sleep measures have been developed to assess sleep patterns and quality of sleep, the concordance of these measures in patients with psychiatric disorders remains relatively elusive.

**Objective:**

This study aims to examine the degree of agreement among 3 sleep recording methods and the consistency between subjective and objective sleep measures, with a specific focus on recently developed devices in a population of individuals with psychiatric disorders.

**Methods:**

We analyzed 62 participants for this cross-sectional study, all having data for polysomnography (PSG), Zmachine, Fitbit, and sleep logs. Participants completed questionnaires on their symptoms and estimated sleep duration the morning after the overnight sleep assessment. The interclass correlation coefficients (ICCs) were calculated to evaluate the consistency between sleep parameters obtained from each instrument. Additionally, Bland-Altman plots were used to visually show differences and limits of agreement for sleep parameters measured by PSG, Zmachine, Fitbit, and sleep logs.

**Results:**

The findings indicated a moderate agreement between PSG and Zmachine data for total sleep time (ICC=0.46; *P*<.001), wake after sleep onset (ICC=0.39; *P*=.002), and sleep efficiency (ICC=0.40; *P*=.006). In contrast, Fitbit demonstrated notable disagreement with PSG (total sleep time: ICC=0.08; wake after sleep onset: ICC=0.18; sleep efficiency: ICC=0.10) and exhibited particularly large discrepancies from the sleep logs (total sleep time: ICC=–0.01; wake after sleep onset: ICC=0.05; sleep efficiency: ICC=–0.02). Furthermore, subjective and objective concordance among PSG, Zmachine, and sleep logs appeared to be influenced by the severity of the depressive symptoms and obstructive sleep apnea, while these associations were not observed between the Fitbit and other sleep instruments.

**Conclusions:**

Our study results suggest that Fitbit accuracy is reduced in the presence of comorbid clinical symptoms. Although user-friendly, Fitbit has limitations that should be considered when assessing sleep in patients with psychiatric disorders.

## Introduction

Sleep disturbances are core comorbid symptoms in psychiatric disorders, with insomnia being one of the predictors of these disorders [1-3]. As sleep disturbances alter prognosis, including relapse and recurrence [4,5], accurately evaluating patient sleep and initiating appropriate treatment early is crucial.

Sleep is evaluated by both subjective and objective measures. Polysomnography (PSG) has been widely accepted as the gold standard for sleep assessment. However, disadvantages include high cost, invasiveness, and difficulty in measuring continuous sleep over long periods [6-8]. Recently, more user-friendly sleep devices have been developed to overcome these problems. For instance, home-based single-channel electroencephalogram (EEG)—the Zmachine Insight Plus—provides algorithm-based sleep staging [9]. Validation studies have indicated the high validity for the sleep parameters and stages, as compared with PSG, not only in people with insomnia but also in patients with psychiatric disorders [10,11]. A consumer wearable device, Fitbit, has also been released, and it is increasingly used in various settings. This device has been reported to have good sensitivity and comparatively poor specificity as compared with PSG in healthy participants and local residents [8,12]. However, few validation studies have been conducted in clinical patients. The relationship between Fitbit and other instruments, such as the single-channel EEG recording or sleep logs, remains elusive, especially in patients with psychiatric disorders. Since these new types of sleep devices are advantageous with regard to both cost and burden [13], identifying concordances and discrepancies between these instruments could provide insight into evaluation methods that could be used for clinical patients. Moreover, our previous studies [10,14] that evaluated the PSG and Zmachine found evidence of a correlation between the severity of obstructive sleep apnea (OSA) and depression with sleep measurements. However, it remains unclear whether these symptoms can influence the association between sleep measurements derived from Fitbit and subjective sleep among patients with various psychiatric disorders.

The aim of our research was (1) to examine the degree of agreements and concordances among the 3 objective methods (PSG, Zmachine, and Fitbit) and sleep logs and (2) to investigate the factors associated with the aforementioned agreements and discrepancies within patients with psychiatric disorders.

## Methods

### Study Participants

Data were collected between the years 2021 and 2022 for this cross-sectional study. All participant data were recorded and collected as part of our clinical care program at Nagoya University Hospital. Inclusion criteria were as follows: individuals who were 18 years of age or older and individuals who had undergone all 4 sleep assessments, including PSG, Zmachine, Fitbit, and sleep logs. Exclusion criteria included individuals who used a continuous positive airway pressure. Trained sleep technologists were responsible for performing the PSG as well as setting up and instructing the patients on the use of Zmachine and Fitbit; they were also responsible for analyzing all the results. Sleep measurement by devices was conducted over a single night to gather data on the sleep patterns of the participants. Before undergoing the sleep recording, all participants completed a self-administered questionnaire and reported on the subjective sleep in the morning following the overnight sleep assessments. Psychiatric diagnosis was made by qualified psychiatrists according to the Diagnostic and Statistical Manual of Mental Disorders, Fifth Edition.

### Ethical Considerations

All study procedures were approved by the Ethics Review Committees at Nagoya University Hospital (approval numbers 2010-0930 and jRCTs042190032), with all participants providing written informed consent in accordance with the Declaration of Helsinki.

### Sleep Measurements

#### Polysomnography

This study uses standard PSGs (Embla N7000 by Natus Neurology Incorporated or PSG-1100 by Nihon Kohden Corporation). The evaluation of the results followed the American Academy of Sleeping Medicine Scoring Manual (version 2.5) [15]. Detailed methods have been described in our prior research [16]. Time in bed (TIB), total sleep time (TST), wake after sleep onset (WASO), sleep efficiency (SE = TST/TIB × 100), and apnea-hypopnea index (AHI) were calculated.

#### Zmachine

The Zmachine Insight Plus (General Sleep Corporation) is an algorithm-based sleep staging EEG device with 1 channel that monitors sleep. Three electrodes were mounted on the mastoids (M1-M2 and EEG signal) and the back of the neck (ground). Zmachine data are stored locally on a microSD card. Zmachine uses its proprietary algorithm to track 3 sleep stages, producing a typical 30-second hypnogram [9,11]. In this study, we evaluated the TST, WASO, and SE (SE = TST/TIB in PSG × 100).

#### Fitbit

We additionally used the Fitbit Sense (Fitbit, Inc) to measure sleep. Fitbit is a wrist-worn activity and heart rate monitor that can also be used as a simple sleep measurement device [17]. Participants wore the Fitbit on a nondominant hand for the whole night. All data were anonymized and uploaded to a secure web server. The algorithm used to interpret the results was the default one provided by the developers, which is neither publicly available nor is it possible to access the raw data generated by the devices. We included TST, WASO, and SE in this study. For SE, the denominator was the recording time measured by the PSG, as described in the method used for Zmachine above.

#### Sleep Logs

Participants were asked to estimate their sleep duration after the monitoring ended in the morning by answering the following questions: (1) “How long did you sleep last night?” and (2) “How long did you stay awake after initially falling asleep?” Subjective TST and WASO corresponded to responses to the first and second questions, respectively. For the SE, as with the other measurements, the PSG TIB was used as the denominator, with the TST divided by the TIB. For responses that had a wide range of estimates, such as 6 to 7 hours, the average was determined and used as the time.

#### Beck Depression Inventory

The Beck Depression Inventory-II (BDI-II) was used to assess the severity of depressive symptoms in this study [18]. The BDI-II is a self-reported measure that consists of 21 items, each of which is rated on a 4-point ordinal response scale that ranges from 0 (indicating the absence of symptoms) to 3 (indicating the most severe symptoms). The BDI-II has been demonstrated to exhibit good psychometric properties, with its total scores ranging from 0 to 63. The BDI-II also provides categorical depression ratings, including “minimal” (0-13), “mild” (14-19), “moderate” (20-28), and “severe” (29-63).

### Statistical Analysis

All results are presented as mean (SD) values. Pearson correlational analysis was performed for the assessment of strength among the 4 sleep measures. We calculated the interclass correlation coefficient (ICC) to evaluate the consistency between sleep parameters obtained from each instrument. In addition to this, Bland-Altman plots were constructed to provide visual agreements or discrepancies between different methods of measurement. To investigate the association with symptoms, we performed an ICC analysis, which excluded severe cases. We defined “severe OSA” as an AHI of 30 or above and “severe depression” as a BDI-II score of 29 or above. For all study analyses, *P*<.05 was considered statistically significant. All statistical analyses were performed using R 4.1.1 (R Core Team) [19].

## Results

### Demographic Characteristics of Participants

**Figure 1:**
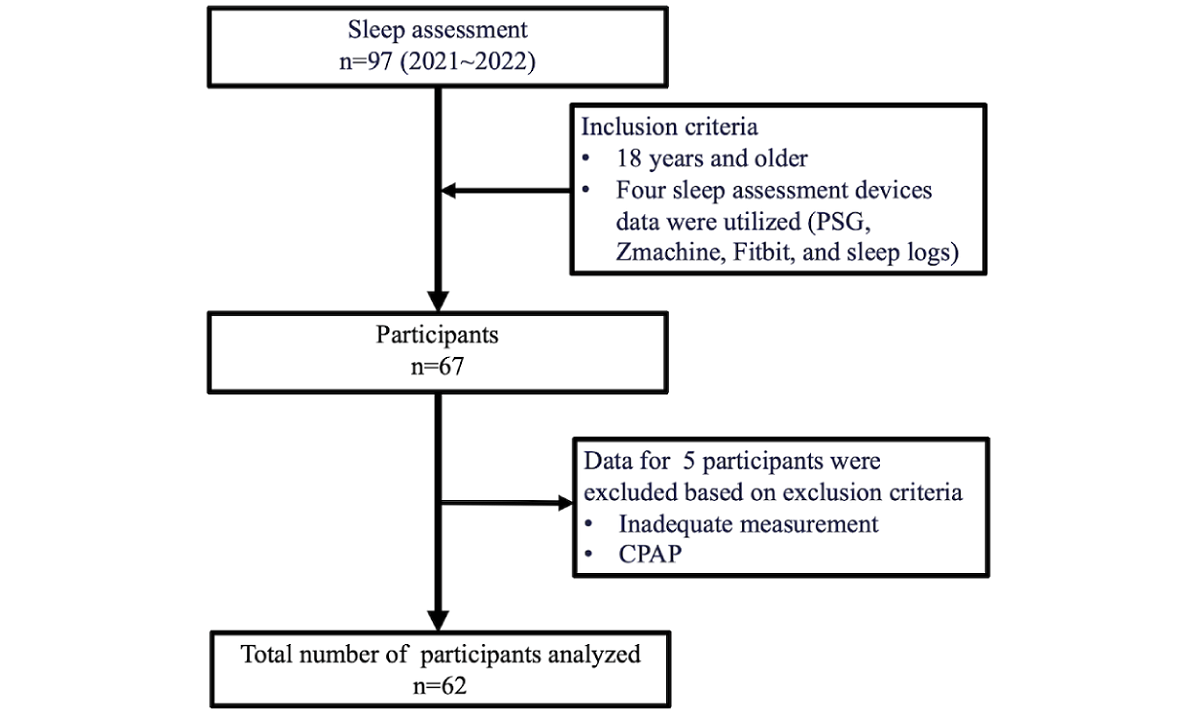
Procedure for participants recruiting. CPAP: continuous positive airway pressure; PSG: polysomnography.

**Figure 2:**
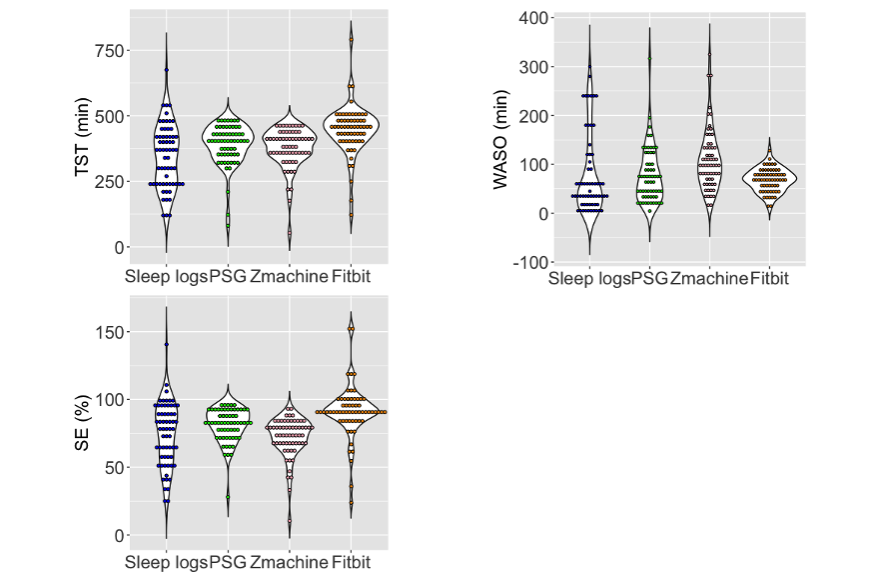
Comparison of sleep parameters for each sleep measure. The y-axis in total sleep time (TST) and wake after sleep onset (WASO) represent minutes, while the y-axis in sleep efficiency (SE) represents percentages. PSG: polysomnography.

Our final analysis evaluated 62 participants (Figure 1). Table 1 presents the characteristics of the participants. Table 2 and Figure 2 display the sleep durations of each instrument. The mean BDI-II score was 20 (SD 11) and the mean AHI value was 17 (SD 21) events/hour. Psychiatric disorder prevalence was as follows: of all the participants, sleep-wake disorders were present in 31% (19/62) of the patients; 21% (13/62) had major depressive disorders; and 15% (9/62) had bipolar disorders. In addition, 13% (8/62) of the participants had neurodevelopmental disorders, while 10% (6/62) had neurocognitive disorders. Moreover, 7% (4/62) of the participants had other psychiatric disorders, such as anxiety disorder and alcoholism. Schizophrenia was present in 5% (3/62) of the sample group. Comorbidities with sleep-wake disorders in the participants included the following: 71% (44/62) had insomnia, 58% (36/62) had OSA, 16% (10/62) had hypersomnia or narcolepsy, and 10% (6/62) exhibited rapid eye movement sleep behavior disorder and periodic limb movements disorder. Furthermore, 2% (1/62) were found to have circadian rhythm disorders.

**Table 1:** Summary of measured variables (N=62).

Variables		Values
Age (years), mean (SD)		46 (18)
**Gender, n (%)**		
	Female	25 (40)
	Male	37 (60)
BMI (kg/m^2^), mean (SD)		24 (5)
Beck Depression Inventory-II, mean (SD)		20 (11)
Apnea-hypopnea index (events/h), mean (SD)		17 (21)
**Psychiatric disorders^a^, n (%)**		
	Sleep-wake disorders	19 (31)
	Major depressive disorders	13 (21)
	Bipolar disorders	9 (15)
	Neurodevelopmental disorders	8 (13)
	Neurodegenerative disease	6 (10)
	Other	4 (7)
	Schizophrenia	3 (5)
**Sleep disorders^b^, n (%)**		
	Insomnia	44 (71)
	Obstructive sleep apnea^c^	36 (58)
	Hypersomnia or narcolepsy	10 (16)
	Rapid eye movement sleep behavior disorder	6 (10)
	Periodic limb movement disorder^d^	6 (10)
	Circadian rhythm disorders	1 (2)

^a^Diagnosis based on the Diagnostic and Statistical Manual of Mental Disorders, Fifth Edition.

^b^Diagnosis based on the International Classification of Sleep Disorders, Third Edition. Some cases overlapped.

^c^Obstructive sleep apnea is defined by an apnea-hypopnea index ≥5 events/hour.

^d^Periodic limb movement disorder is defined by being ≥15.

**Table 2:** Average sleep parameters for each instrument (N=62).

Instrument		Values
**Polysomnography**		
	Total sleep time (min), mean (SD)	389.3 (77.9)
	Wake after sleep onset (min), mean (SD)	76.5 (55.4)
	sleep efficiency (%), mean (SD)	81.4 (12.4)
**Zmachine**		
	Total sleep time (min), mean (SD)	377.3 (76.6)
	Wake after sleep onset (min), mean (SD)	109.3 (63.0)
	sleep efficiency (%), mean (SD)	72.2 (14.8)
**Fitbit**		
	Total sleep time (min), mean (SD)	446.3 (94.5)
	Wake after sleep onset (min), mean (SD)	67.7 (23.3)
	Sleep efficiency (%), mean (SD)	91.2 (19.7)
**Sleep logs**		
	Total sleep time (min), mean (SD)	345.1 (119.4)
	Wake after sleep onset (min), mean (SD)	78.7 (81.9)
	Sleep efficiency (%), mean (SD)	65.7 (23.5)

### Sleep Parameter Agreement

**Figure 3:**
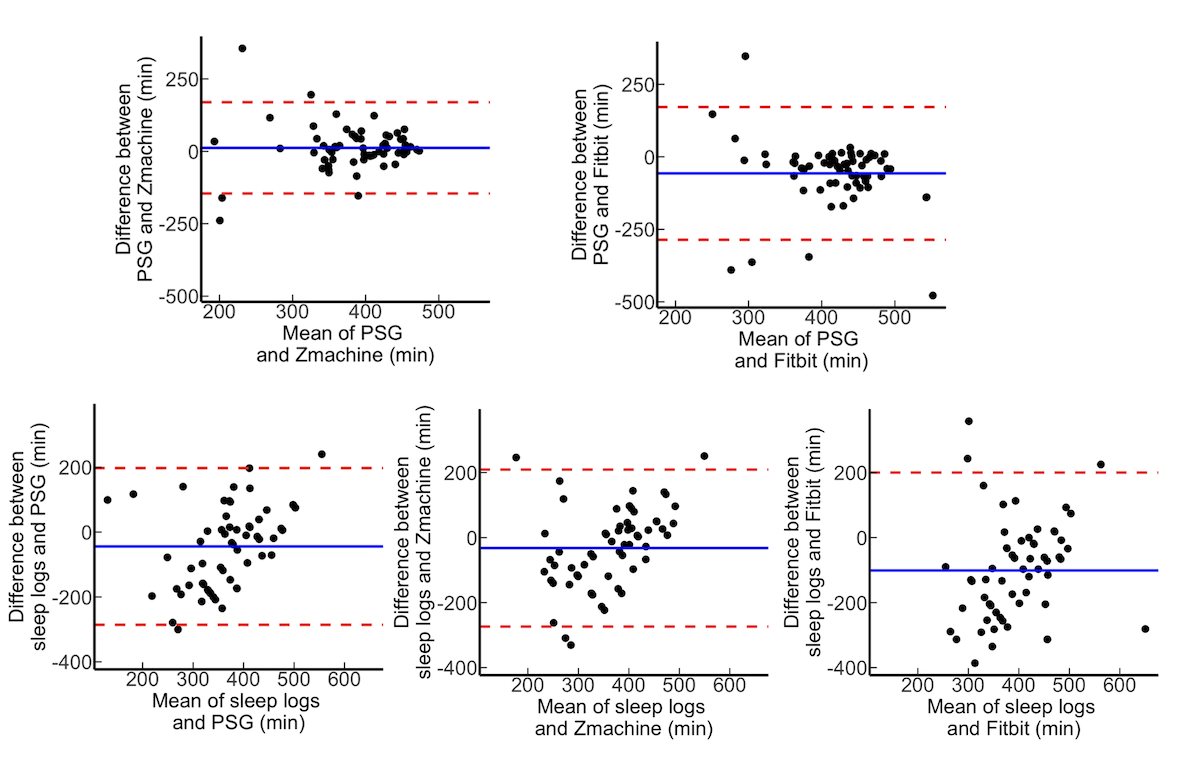
Brand-Altman plot on total sleep time for all participants. PSG: polysomnography.

**Figure 4:**
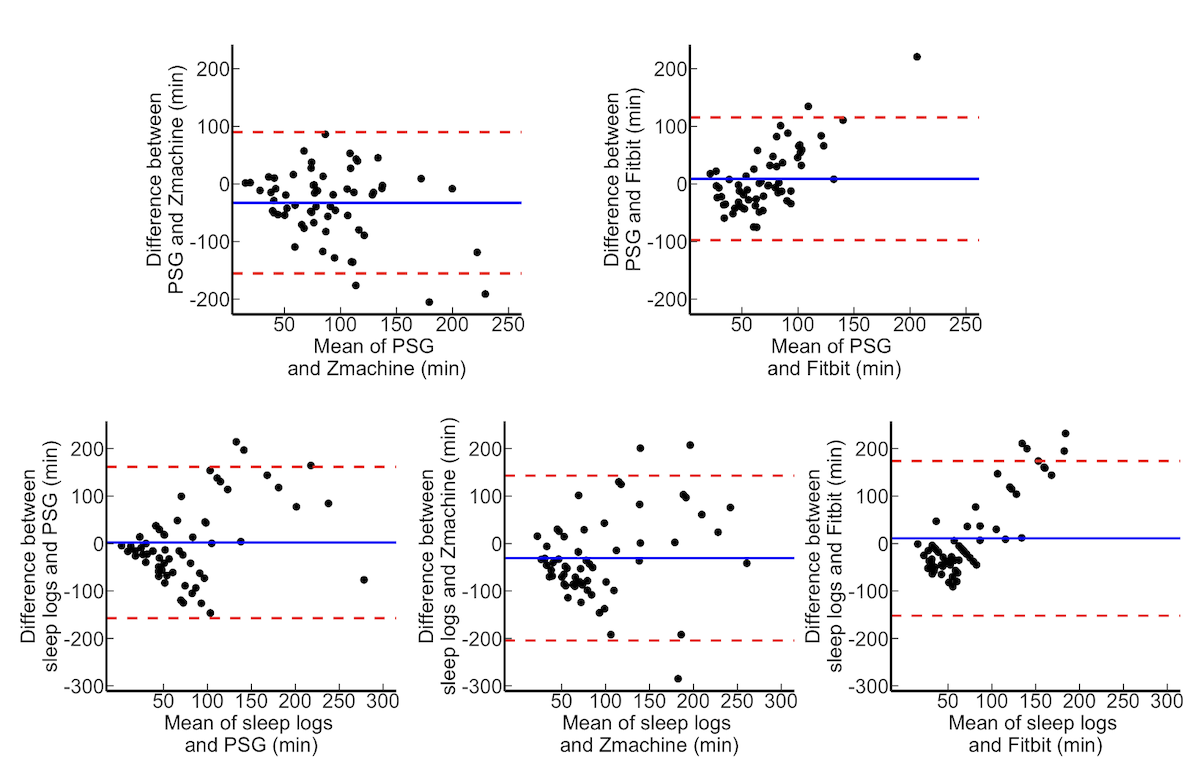
Brand-Altman plot on wake after sleep onset for all participants. PSG: polysomnography.

**Figure 5:**
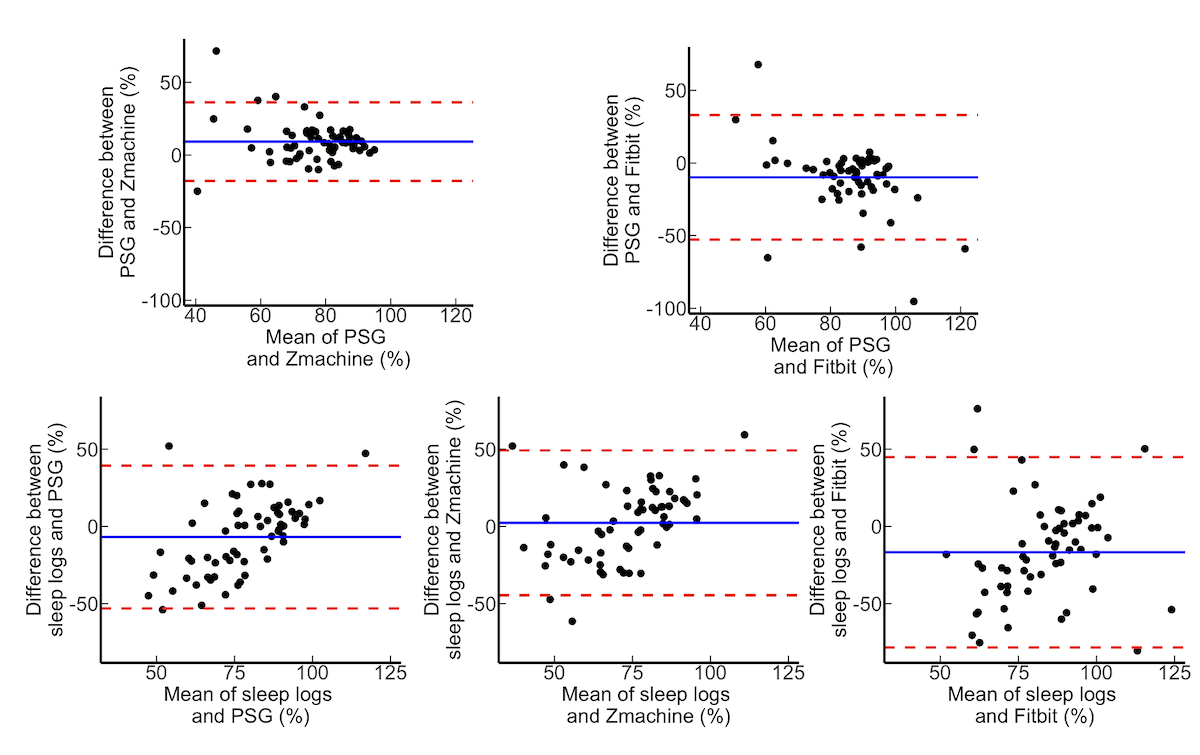
Brand-Altman plot on sleep efficiency for all participants. PSG: polysomnography.

We performed correlation analysis, calculated the ICCs, and conducted Bland-Altman plots to comprehensively confirm the agreement among each measurement (Table 3; Figure 3-5). The TST of the PSG and Zmachine were shown to have a moderate agreement (ICC=0.46) and moderate correlation (*r*=0.46; *P*<.001). The WASO (ICC=0.39; *r*=0.45; *P*<.001) and SE (ICC=0.40; *r*=0.50; *P*<.001) of the PSG and Zmachine exhibited a fair to moderate agreement, which confirmed a moderate correlation. There was no agreement found between the PSG and Fitbit for the TST, WASO, and SE. However, WASO exhibited correlations between the PSG and Fitbit (*r*=0.25; *P*=.047). Although the sleep logs and PSG exhibited a low agreement and significant correlation for the TST (ICC=0.23; *r*=0.28; *P*=.03) and WASO (ICC=0.33; *r*=0.35; *P*=.006), the ICC tended to be relatively low. With regard to the SE between sleep logs and PSG, there was only a weak correlation, with the ICC shown not to be significant (ICC=0.19; *r*=0.31; *P*=.02). The sleep logs and Zmachine exhibited a slight agreement and weak correlation for the TST (ICC=0.24; *r*=0.27; *P*=.03), WASO (ICC=0.24; *r*=0.27; *P*=.03), and SE (ICC=0.24; *r*=0.27; *P*=.03). There were no agreements or correlations between the sleep logs and Fitbit in terms of the TST, WASO, and SE.

**Table 3:** Agreement between sleep parameters measured by polysomnography (PSG), Zmachine, Fitbit, and sleep logs.

Comparison		*r*	*P* value	ICC^a^	*F* test	95% CI (min)	95% CI (max)	*P* value
**Total sleep time**								
	PSG vs Zmachine	0.46	<.001	0.46	2.7	0.24	0.63	<.001
	PSG vs Fitbit	0.09	.47	0.08	1.2	–0.13	0.29	.24
	Sleep logs vs PSG	0.28	.03	0.23	1.68	0.001	0.45	.02
	Sleep logs vs Zmachine	0.27	.03	0.24	1.65	–0.001	0.45	.03
	Sleep logs vs Fitbit	–0.02	.88	–0.01	0.96	–0.18	0.18	.56
**Wake after sleep onset**								
	PSG vs Zmachine	0.45	<.001	0.39	2.59	0.13	0.59	.002
	PSG vs Fitbit	0.25	.047	0.18	1.44	–0.07	0.41	.08
	Sleep logs vs PSG	0.35	.006	0.33	1.95	0.08	0.53	.005
	Sleep logs vs Zmachine	0.27	.03	0.24	1.71	0.01	0.46	.02
	Sleep logs vs Fitbit	0.09	.51	0.05	1.09	–0.21	0.29	.36
**Sleep efficiency**								
	PSG vs Zmachine	0.50	<.001	0.40	2.93	0.09	0.62	.006
	PSG vs Fitbit	0.13	.31	0.10	1.27	–0.11	0.32	.18
	Sleep logs vs PSG	0.31	.02	0.19	1.68	–0.04	0.41	.05
	Sleep logs vs Zmachine	0.27	.03	0.24	1.67	0.004	0.46	.02
	Sleep logs vs Fitbit	–0.04	.79	–0.02	0.93	–0.16	0.15	.61

^a^ICC: interclass correlation coefficient.

### Relation to Symptoms

To examine the relationship between the clinical symptoms and the concordances, a separate ICC analysis was performed by excluding patients with severe OSA and the severe depression, respectively (Multimedia Appendices 1-2). After excluding patients with severe OSA, the ICCs for the TST, WASO, and SE were slightly elevated between the PSG and sleep logs (TST: ICC=0.27; WASO: ICC=0.38; SE: ICC=0.22). There was also an elevation of the ICCs for WASO and SE between the PSG and Zmachine, as compared to before the exclusion of severe OSA (WASO: ICC=0.42; SE: ICC= 0.38). Moreover, when reanalyzed after excluding patients with severe depression, the ICC of the TST for the PSG and sleep logs was marginally elevated (TST: ICC=0.24). All sleep parameters between the sleep logs and Zmachine were slightly higher than those found in all the participants (TST: ICC=0.35; WASO: ICC=0.27; SE: ICC=0.35).

## Discussion

### Principal Findings

This study aimed to compare PSG, Zmachine, Fitbit, and sleep logs in patients with psychiatric disorders and then investigate factors related to the concordances or discrepancies. The comparison of the objective sleep device indicated that there was a low agreement between PSG and Fitbit, even though there was a moderate to high agreement between PSG and Zmachine. Moreover, there was little agreement between the sleep logs and the sleep devices for all sleep parameters, with Fitbit and sleep logs especially exhibiting a remarkable discrepancy. Our results also revealed that depressive symptoms and OSA might potentially be related to the agreement between the subjective and objective sleep measures. However, even after excluding participants with severe OSA and severe depression, we found that there were no sleep parameters that enhanced the ICC between Fitbit and sleep logs. Furthermore, the association between Fitbit and sleep logs exhibited minimal relevance to the aforementioned clinical factors.

None of the sleep parameters recorded by Fitbit exhibited concordance with the PSG, nor did they align with the sleep logs of the individuals with psychiatric disorders. In line with previous research that was conducted on clinical populations, our findings revealed that both TST and SE measurements recorded by Fitbit exhibited an overestimation as compared to PSG, while WASO was observed to be underestimated by Fitbit [20-22]. A study conducted on patients with sleep apnea demonstrated that there was a longer TST for Fitbit, while WASO was shorter when compared to PSG [23]. Similarly, another investigation involving individuals with depression indicated that the TST of Fitbit was longer by 40.6 minutes, with SE overestimated by 7% and WASO 27.1 minutes shorter as compared to PSG [21]. The results of our study implied that Fitbit demonstrated a tendency to overestimate sleep duration while underestimating wakefulness as compared to PSG. In addition, comparisons between Fitbit and sleep logs showed that there were larger discrepancies, with the ICCs not significant for all sleep parameters. Although the underlying mechanism of this discrepancy has yet to be fully understood, it is plausible that the method of sleep stage scoring used by Fitbit could contribute to this discrepancy. Recent models of Fitbit have evaluated sleep patterns by using indicators of body movement and the validity of heart rate measurements, including heart rate variability [20]. Nonetheless, patients with psychiatric disorders often encounter frequent microarousals and sleep fragmentation. People with insomnia, in particular, experience arousal without body movement [24-26]. Additionally, the accurate measurement of the heart rate and monitoring changes in heart rate variability can be challenging in individuals with psychiatric conditions due to the potential decrease in high-frequency activity [27].

A systematic review and meta-analysis [20] of the consistency of PSG and Fitbit revealed a substantial level of validity for sleep parameters, such as TST, WASO, and SE in relation to PSG, particularly in healthy individuals. An empirical study conducted on healthy individuals has provided evidence for the latest model of Fitbit, which is equipped with advanced algorithms [28]. These findings showed the superior performance of the latest model compared to the older models. Furthermore, this latest model demonstrated improved validity compared to PSG [28]. Kang et al [29] reported that the frequency of the acceptable agreement of Fitbit and PSG was significantly lower for the insomnia group as compared to the good-sleepers group, suggesting that the accuracy of Fitbit could be compromised in the presence of clinical symptoms [29]. In terms of agreement with subjective sleep, there was a moderate correlation between the TST of Fitbit and sleep logs among healthy adults [30]. However, the results of our study did not indicate a correlation and concordance between sleep parameters, including for the TST. This discrepancy between subjective sleep and objective sleep measurements, commonly referred to as sleep state misperception, has been observed in various psychiatric disorders [14,31-33]. Findings have shown that this causes adverse effects, including daytime sleepiness, poor quality of subjective sleep, and increased worry about sleep [34,35]. Our study findings suggest that the use of Fitbit as an objective sleep measurement device could exacerbate the incidence of sleep state misperception within these clinical populations. Thus, it is important to use caution when interpreting data from Fitbit in the context of psychiatric disorders, even if Fitbit can provide a relatively reliable assessment of sleep among these healthy populations.

Although our study primally focused on the PSG, Zmachine, Fitbit, and sleep logs, it is essential to acknowledge the prevalent use of actigraphy in evaluating sleep patterns among patients with psychiatric disorders. Previous comparative studies [13,21] have demonstrated significant differences between actigraphy and Fitbit measurements when compared with PSG results. One validation study revealed that actigraphy and Fitbit underestimate TST and overestimate WASO to the same degree. However, increasing the sensitivity of actigraphy brought its measurement closer to PSG [13]. Another study involving patients with depression showed that, compared to PSG, Fitbit with normal settings overestimated TST and SE while underestimating WASO, similar to the findings with actigraphy [21]. Although the direction of these discrepancies is inconsistent across studies, both Fitbit and actigraphy deviate from PSG outcomes to the same degree. The direction and degree of these discrepancies could be dependent on the sensitivity settings of each device and the symptoms of the individuals.

On the other hand, there are clinical situations where Fitbit and actigraphy are useful. Both devices are useful in continuous recording, sleep variability, and circadian rhythm disorders, especially when quick feedback and motivation are needed. In terms of feedback, Fitbit could be superior to actigraphy because of its user-friendly interface [36]. It has also been suggested that Fitbit is more useful for measuring sleep onset latency in patients with depression [21]. Because actigraphy can be used for extended periods of time, it is more advantageous than Fitbit for identifying circadian rhythm disorders [37]. Thus, clinicians should consider the characteristics of wearable devices and determine their indications.

Each sleep parameter measured by PSG and Zmachine demonstrated a higher consistency, which is in line with previous studies [10]. Previous research that studied the accuracy of PSG and Zmachine in patients with insomnia demonstrated good validity for each sleep parameter and stage [9,11]. Our previous study in patients with psychiatric disorders also showed significant agreements with the PSG in terms of TST, WASO, and SE [10]. With regard to PSG or Zmachine and the sleep logs, the ICCs demonstrated low agreement, ranging from 0.24 to 0.37, which is partially significant on sleep parameters. When severe depressive symptoms and OSA were excluded, the ICCs for subjective and objective sleep, as measured by PSG and Zmachine, showed an increase, albeit slightly, suggesting that these symptoms may have some impact on these discrepancies. We previously reported finding associations between depression and OSA, which are related to sleep state misperception in patients with depression [14].

### Strengths and Limitations

The strength of our study is the use of real clinical data from patients with psychiatric disorders to assess the consistency between multiple sleep instruments, including sleep logs and commercially available sleep equipment. However, there are several limitations associated with our study. First, the sample size of this study was relatively small. Second, we could not conduct a subgroup analysis according to the psychiatric and sleep disorders. Previous research has reported that the identification of psychiatric and sleep problems resulted in variations in sleep architectures [3]. Third, we only assessed sleep for 1 night. We successfully highlighted the characteristics of each measurement method by comparing each sleep measurement, but we did not consider the advantage of the long-term tracking features of wearable devices. To overcome these limitations, future research will need to investigate the measurement method affinity for each of the psychiatric and sleep disorders when using a larger sample size, including other sleep measurement devices for multiple days.

### Conclusions

The outcomes obtained through the use of Fitbit revealed inconsistencies in the comparisons of the measurements derived from PSG and sleep logs in individuals with psychiatric disorders. In terms of the relationship with subjective sleep, the use of Fitbit devices has the potential to induce sleep state misperception. Although the use of Fitbit in sleep assessment has been expanding, it is crucial that its limitations are recognized when evaluating patients with psychiatric disorders.

## Appendix

Multimedia Appendix 1 Agreement between sleep parameters measured by polysomnography, Zmachine, Fitbit, and sleep logs, after excluding patients with severe obstructive sleep apnea.

Multimedia Appendix 2 Agreement between sleep parameters measured by polysomnography, Zmachine, Fitbit, and sleep logs, after excluding patients with severe depression.

## Abbreviations

AHI: apnea-hypopnea index
BDI-II: Beck Depression Inventory-II
EEG: electroencephalogram
ICC: interclass correlation coefficient
OSA: obstructive sleep apnea
PSG: polysomnography
SE: sleep efficiency
TIB: time in bed
TST: total sleep time
WASO: wake after sleep onset

## Data Availability

The data sets generated and analyzed during this study are available from the corresponding author on reasonable request.
