# Supplementary material for: Comparison of Polysomnography, Single-Channel Electroencephalogram, Fitbit, and Sleep Logs in Patients With Psychiatric Disorders: Cross-Sectional Study
Source: J Med Internet Res. 2023 Dec 13;25:e51336. doi: 10.2196/51336 (PMC10753421; doi:10.2196/51336)
Supplement: Multimedia Appendix 1 [file jmir_v25i1e51336_app1.docx]

| **comparison** | ***r*** | ***P* value** | **ICC** | **F (df)** | **95%CI (min)** | **95%CI (max)** | ***P* value** |
| --- | --- | --- | --- | --- | --- | --- | --- |
| **TST** |  |  |  |  |  |  |  |
| P vs. Z | 0.38 | .007 | 0.38 | 2.22 | 0.11 | 0.60 | .003 |
| P vs. F | 0.03 | .83 | 0.03 | 1.06 | -0.20 | 0.27 | .42 |
| S vs. P | 0.32 | .02 | 0.27 | 1.82 | 0.01 | 0.51 | .02 |
| S vs. Z | 0.27 | .06 | 0.23 | 1.62 | -0.04 | 0.47 | .047 |
| S vs. F | -0.05 | .76 | -0.03 | 0.92 | -0.22 | 0.19 | .62 |
| **WASO** |  |  |  |  |  |  |  |
| P vs. Z | 0.47 | <.001 | 0.42 | 2.77 | 0.15 | 0.63 | .002 |
| P vs. F | 0.30 | .04 | 0.22 | 1.55 | -0.07 | 0.47 | .07 |
| S vs. P | 0.39 | .006 | 0.38 | 2.18 | 0.10 | 0.59 | .004 |
| S vs. Z | 0.22 | .12 | 0.20 | 1.55 | -0.06 | 0.44 | .07 |
| S vs. F | -0.02 | .91 | -0.01 | 0.98 | -0.29 | 0.27 | .53 |
| **SE** |  |  |  |  |  |  |  |
| P vs. Z | 0.46 | <.001 | 0.38 | 2.69 | 0.07 | 0.61 | .008 |
| P vs. F | 0.26 | .07 | 0.22 | 1.66 | -0.03 | 0.46 | .04 |
| S vs. P | 0.35 | .01 | 0.22 | 1.78 | -0.04 | 0.46 | .048 |
| S vs. Z | 0.27 | .06 | 0.22 | 1.60 | -0.05 | 0.47 | .05 |
| S vs. F | 0.0006 | .99 | 0.0004 | 1.00 | -0.16 | 0.20 | .50 |

Abbreviations: TST, total sleep time; WASO, wake after sleep onset; SE, sleep efficiency; P, polysomnography; Z, Zmachine; F, Fitbit; S, sleep logs; OSA obstructive sleep apnea.
