# Supplementary material for: Comparison of Polysomnography, Single-Channel Electroencephalogram, Fitbit, and Sleep Logs in Patients With Psychiatric Disorders: Cross-Sectional Study
Source: J Med Internet Res. 2023 Dec 13;25:e51336. doi: 10.2196/51336 (PMC10753421; doi:10.2196/51336)
Supplement: Multimedia Appendix 2 [file jmir_v25i1e51336_app2.docx]

| **comparison** | ***r*** | ***P* value** | **ICC** | **F(df)** | **95%CI (min)** | **95%CI (max)** | ***P* value** |
| --- | --- | --- | --- | --- | --- | --- | --- |
| **TST** |  |  |  |  |  |  |  |
| P vs. Z | 0.38 | .009 | 0.38 | 2.24 | 0.10 | 0.60 | .004 |
| P vs. F | 0.04 | .81 | 0.03 | 1.07 | -0.21 | 0.29 | .41 |
| S vs. P | 0.27 | .07 | 0.24 | 1.65 | -0.04 | 0.50 | .048 |
| S vs. Z | 0.37 | .01 | 0.35 | 2.07 | 0.07 | 0.59 | .009 |
| S vs. F | -0.01 | .95 | -0.01 | 0.98 | -0.23 | 0.24 | .53 |
| **WASO** |  |  |  |  |  |  |  |
| P vs. Z | 0.36 | .02 | 0.28 | 2.03 | 0.006 | 0.52 | .02 |
| P vs. F | 0.23 | .13 | 0.18 | 1.44 | -0.12 | 0.45 | .11 |
| S vs. P | 0.35 | .02 | 0.32 | 1.94 | 0.03 | 0.56 | .02 |
| S vs. Z | 0.33 | .03 | 0.27 | 1.99 | -0.002 | 0.52 | .03 |
| S vs. F | 0.10 | .53 | 0.06 | 1.11 | -0.25 | 0.34 | .36 |
| **SE** |  |  |  |  |  |  |  |
| P vs. Z | 0.45 | .002 | 0.32 | 2.42 | -0.002 | 0.57 | .03 |
| P vs. F | 0.07 | .65 | 0.05 | 1.11 | -0.20 | 0.31 | .36 |
| S vs. P | 0.34 | .02 | 0.21 | 1.68 | -0.05 | 0.45 | .06 |
| S vs. Z | 0.37 | .01 | 0.35 | 2.06 | 0.06 | 0.58 | .009 |
| S vs. F | -0.03 | .86 | -0.02 | 0.95 | -0.20 | 0.20 | .57 |

Abbreviations: TST, total sleep time; WASO, wake after sleep onset; SE, sleep efficiency; P, polysomnography; Z, Zmachine; F, Fitbit; S, sleep logs; OSA obstructive sleep apnea.
